# The Dual Role of Sulforaphane-Induced Cellular Stress—A Systems Biological Study

**DOI:** 10.3390/ijms25021220

**Published:** 2024-01-19

**Authors:** Marianna Holczer, Boglárka Besze, Annamária Lehel, Orsolya Kapuy

**Affiliations:** Department of Molecular Biology, Institute of Biochemistry and Molecular Biology, Semmelweis University, 1085 Budapest, Hungary; holczer.marianna@semmelweis.hu (M.H.); bebogi94@gmail.com (B.B.); lehelpanni@gmail.com (A.L.)

**Keywords:** autophagy, sulforaphane, feedback loops, cellular stress, systems biology

## Abstract

The endoplasmic reticulum (ER) plays a crucial role in cellular homeostasis. When ER stress is generated, an autophagic self-digestive process is activated to promote cell survival; however, cell death is induced in the case of excessive levels of ER stress. The aim of the present study was to investigate the effect of a natural compound called sulforaphane (SFN) upon ER stress. Our goal was to investigate how SFN-dependent autophagy activation affects different stages of ER stress induction. We approached our scientific analysis from a systems biological perspective using both theoretical and molecular biological techniques. We found that SFN induced the various cell-death mechanisms in a concentration- and time-dependent manner. The short SFN treatment at low concentrations promoted autophagy, whereas the longer treatment at higher concentrations activated cell death. We proved that SFN activated autophagy in a mTORC1-dependent manner and that the presence of ULK1 was required for its function. A low concentration of SFN pre- or co-treatment combined with short and long ER stress was able to promote cell survival via autophagy induction in each treatment, suggesting the potential medical importance of SFN in ER stress-related diseases.

## 1. Introduction

Cellular systems are affected by several external and internal stimuli that can trigger various stress responses mechanisms. Different signaling pathways can be activated depending on the type of stress (e.g., heat shock response, unfolded protein response, etc.) to turn on either survival or cell-death processes. The outcome of the response directly depends on the intensity and duration of the stress. Specifically, in response to stress, cells try to return to their previous homeostatic state, but if this is not possible, cell-death processes are induced to eliminate the damaged cell [1,2,3]. The most frequently activated processes are autophagy, apoptosis, and necroptosis [1,4]. Autophagy is a so-called self-eating mechanism, where double-membrane vesicles are formed, and the content of these vesicles is broken down. Then, the cell can use these released building blocks to improve its survival [5,6]. However, permanent autophagy can cause the death of the cell, too [4,7]. Apoptosis is the well-known suicide mechanism of damaged or redundant cells, where the cell breaks down into apoptotic bodies [4,8]. During necroptosis, the organelles swell, followed by their lysis, which results in the release of intracellular substances into the environment followed by an inflammatory process [4,8].

Cells try to deal with the different types of stress events in different ways [2]. For example, if proteins with bad conformation accumulate in the lumen of the endoplasmic reticulum for any reason (e.g., nutrient deficiency, imbalance in Ca^2+^ metabolism, toxin effect, oxidative stress, viral infection, mutant protein), the homeostasis of the organelle becomes disrupted, and a series of self-defense processes for survival are initiated, which is called ER stress-response mechanism [9]. ER stress-response mechanism directly activates the unfolded protein response (UPR) signaling pathway [10]. The main goal of UPR is protective by restoring ER homeostasis, promoting the re-folding or degradation of proteins through the induction of chaperones and enzymes involved in folding and reducing the load on the organelle by stopping de novo protein synthesis [10,11]. However, UPR can promote the activation of apoptotic and necrotic cell death under severe or prolonged stress [11,12].

The connection between ER stress and autophagy is essential in the maintenance of cellular homeostasis, and therefore, it has great importance in investigating the causes and treatment of certain diseases [13,14]. In inflammatory disorders and neurodegenerative diseases, there is severe ER stress in the cell that induces apoptosis. However, the activation of autophagy via UPR can prevent cell death, and the digestion of damaged components can increase the survival of the cells. In contrast, during cancers, the cells can abolish the negative effect of ER stress with the help of autophagy and extend their lifespan [13,15]. Therefore, all those compounds, which can affect autophagy (activation or inhibition), might have a crucial role in the therapy of these diseases [13,16].

Previously, our group has made some mathematical models to analyze the ER stress-response mechanism [17,18], focusing on the crosstalk between autophagy and apoptosis [19,20] and the autophagy-dependent survival mechanism under various types of cellular stress [21,22,23,24]. We have found that autophagy can be periodically activated due to the delayed negative feedback loop between AMPK and ULK1 [22,25]. Furthermore, we have claimed that a robust autophagy response mechanism requires double-negative and positive feedback loops in the system [24]. We have previously confirmed that the mutual antagonism between AUTA effector (autophagy effector) and APOA effector (apoptosis effector) guarantees that autophagy and apoptosis cannot be active at the same time with respect to ER stress [18,19,20].

The activation of autophagy can be promoted using various natural compounds (such as resveratrol and epigallocatechin gallate (EGCG)) so that even in the case of severe ER stress, the activation of apoptosis can be delayed in time [26,27]. Resveratrol (trans-3,5,4 ${\;}^{\prime }$ -trihydroxystilbene) is a natural polyphenol compound found in the roots of plants, berries, and grapes. It can inhibit mTORC1 through activation of AMPK and is also able to inhibit mTORC1 directly in an ATP-competitive manner, thus affecting the mTORC1-ULK1 pathway and activation of autophagy [28,29]. EGCG is a flavon-3-ol phenolic compound found in green tea [30,31]. It can enhance Beclin-1 expression, ATG5, LC3B [32] and AMPK activity [33,34], and is able to inhibit AKT/STAT3 pathway [32] and mTORC1 [33,35]. Sulforaphane (SFN) is an isothiocyanate, and its precursor, glucoraphanin, is naturally found in some cruciferous vegetables like broccoli and cabbage [36]. It has cytoprotective, anti-oxidant, anti-cancer, and anti-inflammatory properties [16,36]. Based on the results so far, the effect of SFN is not completely clear, as it was able to both activate and inhibit autophagy, promote apoptosis, induce ER stress, and inhibit or not mTORC1 [37,38,39,40]. However, the role of SFN, namely its ability to increase cell survival under ER stress, is not yet proven.

In the present study, we explored the role of SFN treatment using a systems biology perspective. We built up a tiny molecular network of ER stress-response mechanisms based on ordinary differential equations to investigate the effect of SFN further. Moreover, we performed some essential molecular biology experiments to verify and improve the performance of our model. At the end of these processes, our model was able to describe the dynamic behavior of SFN-related ER stress-response mechanisms and predict the outcome of new experiments.

## 2. Results

### 2.1. SFN Induces Cell Death Both in Time- and Concentration-Dependent Manner

**Figure 1:**
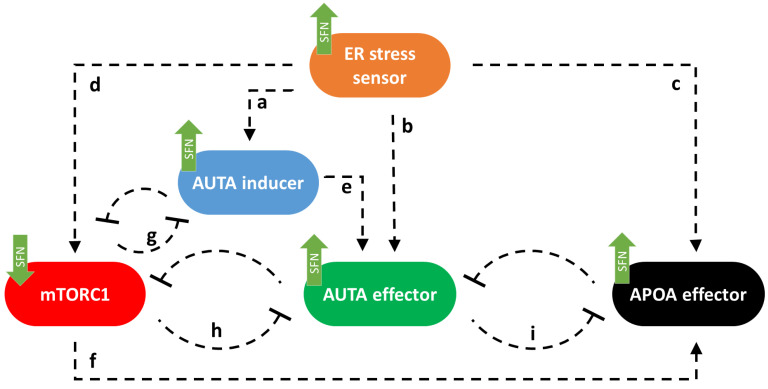
The wiring diagram of endoplasmic reticulum (ER) stress-response mechanism. The ER stress sensor, the autophagy inducer (AUTA inducer), the mTORC1, the autophagy effector (AUTA effector), and the apoptosis effector (APOA effector) are denoted by isolated orange, blue, red, green, and black boxes, respectively. Dashed lines show how the components can influence each other, while blocked end lines denote inhibition. The green arrows show how sulforaphane acts in this system, according to data from the literature and a–i indicate connections between the components.

First, we built up a simple regulatory network of cellular life-and-death decisions upon endoplasmic reticulum stress, paying particular attention to the attack points of sulforaphane. Corresponding to our previous study [23], here the ER stress-response mechanism can be described with the following wiring diagram of four regulators, these are called ER stress sensor, AUTA inducer, mTORC1, AUTA effector, and APOA effector, respectively (Figure 1). ER stress sensor includes all those molecules that can be induced directly by the ER stress-response mechanism like PERK, IRE1, and ATF6 [10]. These molecules play an important role in relaying the signal to the executors and, thus, in promoting the cellular response. We claim that this ER stress sensor can induce both cellular survival and death by activating the AUTA inducer (a in Figure 1) [10,41,42], the AUTA effector (b in Figure 1) [10,42,43], the APOA effector (c in Figure 1) (e.g., caspase3 (Casp3), and other caspases) [12,44,45] and the mTORC1 (d in Figure 1) [46] depending on the degree and the length of the stress [2,20,47]. We assume that the group of AUTA inducers contains all those molecules which are able to promote the formation of autophagy activator complex (e.g., AMPK, ULK1), while we call a molecule AUTA effector if it is able to induce autophagy via helping the activation of the autophagosome formation (e.g., Beclin1). The AUTA inducer promotes autophagy via the AUTA effector (e in Figure 1), while the APOA effector induces apoptosis. Similarly, the APOA effector contains all those molecules that are able to promote apoptotic cell death (Figure 1).

Corresponding to already published data we assume that mTORC1 becomes downregulated by both AUTA inducer (g in Figure 1) [48,49] and AUTA effector (h on Figure 1) [26,50], while mTORC1 can inhibit both the AUTA inducer (g in Figure 1) [21,51,52] and the AUTA effector (h in Figure 1) [53,54,55] and it can influence APOA effector positively (f in Figure 1) [56], too. The double-negative feedback loops between AUTA inducer and mTORC1 (g) and between AUTA effector and mTORC1 (h) contribute to ensuring that autophagy is only activated when it is needed [21,23]. Between AUTA effector and APOA effector, there is a mutual inhibition (i in Figure 1) [20,23,57] (Figure 1).

It has already been shown that sulforaphane (SFN) could disturb the ER stress-response mechanism [38]. Meanwhile it was able to inhibit mTORC1 [39] and activate both autophagy [36,37,38] and apoptosis [58,59]. Furthermore, autophagy inactivation was also observed with SFN treatment [39,58]. Here, we assumed that SFN could induce the activation of the ER stress sensor, AUTA inducer, AUTA effector, and APOA effector. Meanwhile, it had a negative effect on mTORC1 (see green arrows in Figure 1).

**Figure 2:**
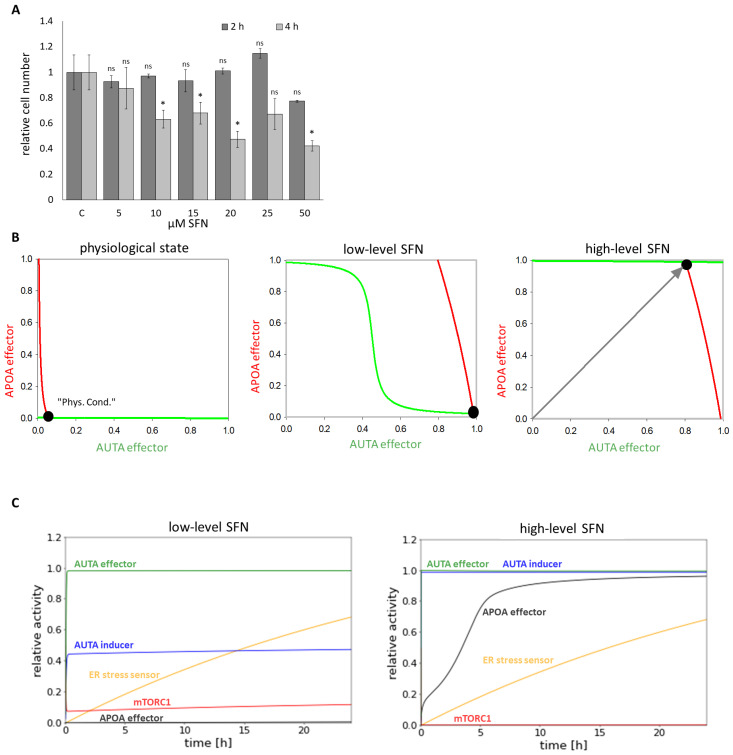
The time- and concentration-dependent effect of the sulforaphane on the viability of the cells. HEK293T cells were treated with 5, 10, 15, 20, 25, and 50 μ M SFN for 2 and 4 h meanwhile (**A**) the relative number of viable cells was denoted. Error bars represent standard deviation. Asterisks indicate statistically significant differences from the control: *ns*—non significant; *—*p* < 0.05. (**B**) Phase plane diagram of endoplasmic reticulum (ER) stress-response mechanism under physiological state (left panel), at low (middle panel) (parameter setting for the simulation: SFN = 1, stress = 10) and at high (right panel) (parameter setting for the simulation: SFN = 80, stress = 10) level of SFN. The balance curves of AUTA effector (green curve) and APOA effector (red curve) are plotted. Stable steady states are visualized with black dots. (**C**) The computational simulations are determined upon low (left panel) (parameter setting for the simulation: SFN = 1, stress = 10) and high (right panel) (parameter setting for the simulation: SFN = 80, stress = 10) SFN treatments. The relative activity of mTORC1, AUTA inducer, ER stress sensor, APOA effector, and AUTA effector is shown.

To further investigate the role of SFN upon cellular stress, first human embryonic kidney cells (HEK293T) were treated with 5, 10, 15, 20, 25, and 50 μ M concentrations of SFN for 2, 4, and 24 h, respectively. Then, we examined the relative number of viable cells (Figure 2A and Figure S1). The number of living cells was not decreased in the short treatment (2 h) up to a concentration of 50 μ M and in the longer treatments (4 and 24 h) at low concentrations (5 μ M), suggesting that SFN did not have a harmful effect on the cells (Figure 2A and Figure S1). However, corresponding to the already published data [38,39], we could demonstrate that either increasing concentrations of SFN or increasing duration of treatment reduced the number of living cells (Figure 2A and Figure S1).

To investigate the dynamical characteristic of the stress-response mechanism induced by SFN, a so-called signal response curve was plotted, where the activity of both AUTA and APOA effectors was followed upon SFN treatment (Figure 2B). The coordinate system was spanned by AUTA and APOA effectors, and the so-called nullclines—namely dAUTA effector/dt = 0 (green) and dAPOA effector/dt = 0 (red)—were depicted. Where the nullclines intersected each other, the system could be in a steady state. These can be the states of the biological system that can be observed at certain conditions. Under physiological conditions, the stress signal was zero. In this case, there was only one stable state, called “Phys.Cond”. with low levels of both AUTA and APOA effectors (Figure 2B, panel left). At a low level of SFN (which was taken into account in the model by increasing the level of SFN from 0 to 1 and stress from 0 to 10), the nullcline of APOA effector moved to the right, resulting in a stable intersection when AUTA effector was active, but APOA was inactive (Figure 2B, middle panel). Interestingly, as the SFN level increased (i.e., 80X more SFN than before) the nullcline of AUTA effector moved upward, suggesting a stable state where both autophagy and apoptosis were active in the cell (Figure 2B, panel right).

To further analyze the role of SFN treatment, time-course simulations were also performed both at a low and high level of sulforaphane (Figure 2C). We claimed that autophagy and apoptosis are active when 75% of AUTA and APOA effectors became active. Our results showed that at low-level SFN, autophagy was able to turn on, while mTORC1 and apoptosis remained inactive (Figure 2C, panel left). In contrast, at high-level SFN, both AUTA inducer and AUTA effector were active, but a rapid activation of APOA effector was also seen, suggesting that the self-cannibalism could not work properly; rather, cells committed suicide (Figure 2C, panel right).

Our systems biology analysis supposes that a low level of SFN does not decrease the number of viable cells. Meanwhile, the treatment turns on autophagy; however, excessive levels of SFN enhance apoptotic cell death, but autophagy also remains active.

### 2.2. In Contrast to SFN-Induced Apoptosis, SFN-Promoted Autophagy Does Not Decrease the Lifespan of Cells

Our theoretical analysis suggested that autophagy became activated at low SFN concentrations, and according to the viability assay, no cell death was observed when only autophagy was active, whereas apoptosis was induced at high concentrations after about 4 h. Data have already suggested that SFN induced ER stress in cells [38]. Therefore, here, we investigated the role of SFN in autophagy and apoptosis regulation, directly focusing on its role in ER stress induction.

**Figure 3:**
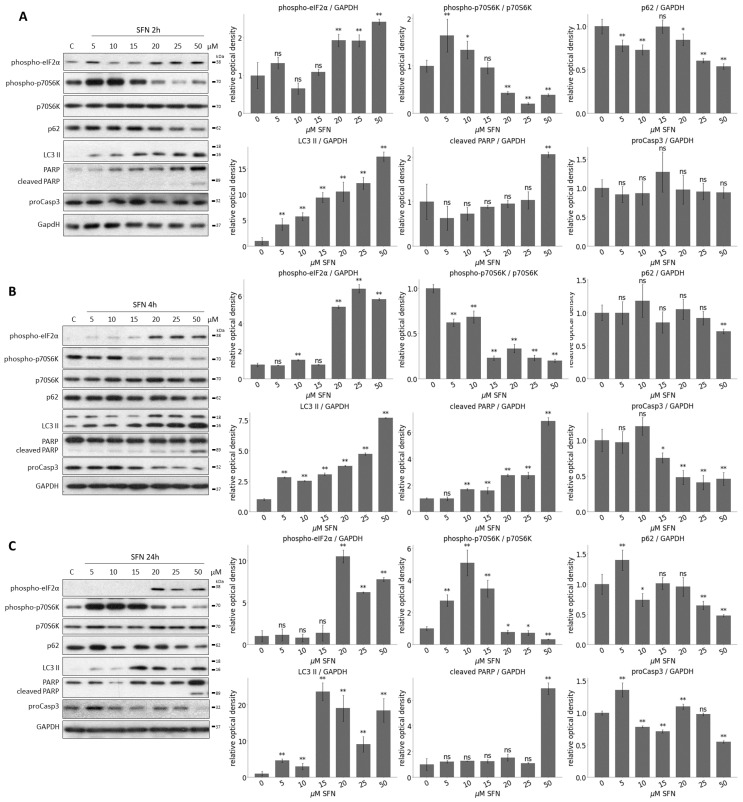
The time- and concentration-dependent effect of the sulforaphane on the members of the control network. HEK293T cells were treated with 5, 10, 15, 20, 25, and 50 μ M SFN for (**A**) 2, (**B**) 4, and (**C**) 24 h. The markers of ER stress (phospho-eIF2 α ), the markers of mTORC1 (phospho-p70S6K), the markers of autophagy (p62, LC3 II) and apoptosis (cleaved PARP, proCasp3) were followed by immunoblotting. GAPDH was used as a loading control (left panels). Densitometry data represent the intensity of (phospho-p70S6K) normalized for the total level of p70S6K, (phospho-eIF2 α ), p62, LC3 II, cleaved PARP and proCasp3 normalized for GAPDH (right panel). Error bars represent standard deviation. Asterisks indicate statistically significant differences from the control: *ns*—non significant; *—*p* < 0.05; **—*p* < 0.01.

To detect the activation profile or levels of the key members of the regulatory network during SFN treatments, immunoblotting was performed (Figure 3 and Figure S2). The activation of ER stress (phospho-eIF2 α (eukaryotic initiation factor 2)), the activity of mTORC1 (phospho-p70S6K), and the activation of autophagy (e.g., p62, LC3 II) and apoptosis (cleaved PARP, procaspase3 (proCasp3)) were monitored during SFN treatment with different concentrations at three different time points, 2 h (Figure 3A and Figure S2), 4 h (Figure 3B) and 24 h (Figure 3C). EIF2 α phosphorylation is an early ER stress marker, so the increase in phospho-eIF2 α levels showed that the cells were under ER stress during the 2 h SFN treatment (Figure 3A). However, for the 4 and 24 h treatments, the phosphorylation of eIF2 α was significantly increased at 20 μ M SFN, and this phosphorylation was also maintained at higher concentrations so that ER stress increased further in cells with increasing concentrations of SFN (Figure 3B,C).

The activity of mTORC1 was inferred from the phosphorylation state of its downstream target, p70S6K. When mTORC1 is active, it can phosphorylate p70S6K. As a result of the treatments, phosphorylation of p70S6K started to decrease at 15–20 μ M of SFN, from which point mTORC1 seemed to be inactivated. This inactivation was maintained until the end of the treatments (Figure 3 and Figure S2).

The induction of intensive autophagy was observed after 15 μ M SFN concentration (see decreasing of p62 and increasing of LC3 II in Figure 3 and Figure S2). No cleavage was observed for PARP, and the level of proCasp3 did not decrease, so even at higher concentrations, apoptosis was not observed in cells after 2 h of treatment of SFN (Figure 3A and Figure S2). In contrast, under 4 and 24 h SFN treatment, the cleaved form of PARP appeared at concentrations of 50 μ M, suggesting that high SFN concentrations induced apoptotic processes. This was also shown by the gradually decreasing levels of proCasp3, which disappeared completely at 50 μ M after 24 h (Figure 3B,C).

Taken together, we assume that a short treatment with low concentrations of SFN promotes autophagy-dependent survival, whereas a longer treatment at higher concentrations prefers apoptotic cell death. Meanwhile, autophagy remains active, too.

### 2.3. SFN-Induced Autophagy Requires ULK1 and Acts via mTORC1 Pathway

**Figure 4:**
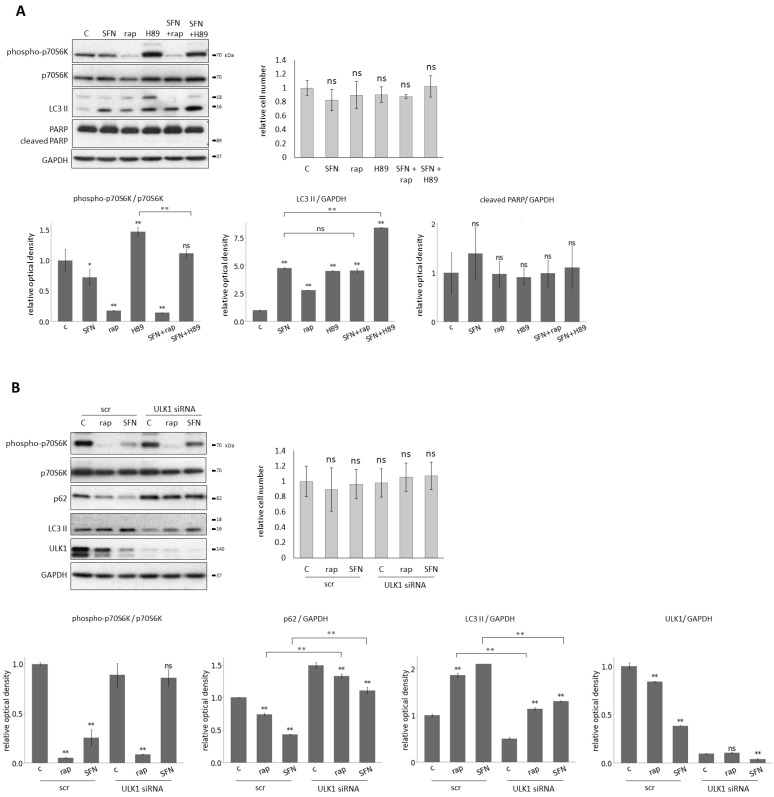
Sulforaphane induces autophagy through mTORC1 pathway with the help of ULK1. HEK293T cells were treated with 15 μ M SFN for 2 h, 100 nM rapamycin (rap) for 2 h, and 2.5 nm H89 for 2 h. ULK1 was silenced with siRNA in the cells and scrambled siRNA (scr) was used as a negative control. (**A**) The combined treatment with rap and H89. The markers of mTORC1 (phospho-p70S6K), autophagy (LC3 II), and apoptosis (cleaved PARP) were followed by immunoblotting. GAPDH was used as a loading control (left panel). The relative number of viable cells was denoted (right panel). Densitometry data represent the intensity of phospho-p70S6K normalized for the total level of p70S6K, LC3 II, and cleaved PARP normalized for GAPDH (lower panel). (**B**) The combined treatment with ULK1 silencing. The markers of mTORC1 (phospho-p70S6K), autophagy (p62, LC3 II) and ULK1 were followed by immunoblotting. GAPDH was used as a loading control (left panel). The relative number of viable cells was denoted (right panel). Densitometry data represent the intensity of phospho-p70S6K normalized for the total level of p70S6K, p62, LC3 II, and ULK1 normalized for GAPDH (lower panel). Error bars represent standard deviation. Asterisks indicate statistically significant differences from the control: *ns*—non significant; *—*p* < 0.05; **—*p* < 0.01.

To further explore the characteristic of SFN-induced autophagy, we have tested its effects in combination with various drugs that induce autophagy through different pathways: one of them via downregulation of mTORC1 (rapamycin (rap)) [60,61] and the other through inhibition of PKA independently from mTORC1 (H89) [29] (Figure 4). Thus, to understand the mechanism of SFN-induced autophagy, HEK293T cells were treated with either rap (100 nM, 2 h) or H89 (2.5 μ M, 2 h) and SFN (15 μ M, 2 h) without/with followed by rap (100 nM, 2 h) or H89 (2.5 μ M, 2 h) addition.

These treatments did not cause any decrease in the relative number of viable cells, and this was consistent with the fact that no PARP cleavage was observed (Figure 4A). Next, one of the key markers of mTORC1, autophagy, and apoptosis was detected by immunoblotting (Figure 4A). There was neither additive effect on autophagy induction nor mTORC1 inhibition by the rap+SFN combined treatment, suggesting that both SFN and rap acted via the same pathway to induce autophagy. In contrast, the H89+SFN combined treatment had a significant additive effect on autophagy activation and mTORC1 inhibition, indicating that SFN and H89 used different pathways to promote autophagy (Figure 4A).

It is well-known that the key inducer of autophagy is ULK1. Meanwhile, mTORC1 inhibits it in a phosphorylation-dependent manner under physiological conditions [62,63]. Since SFN could induce autophagy via downregulation of mTORC1, we also investigated whether or not ULK1 was required for autophagy induction upon SFN treatment. We carried out SFN (15 μ M, 2 h) and rap treatments (100 nM, 2 h) in the presence or absence of ULK1 (Figure 4B). ULK1 knock-down itself using siULK1 did not affect the relative number of viable cells (Figure 4B), suggesting that no cell death was detected. However, when SFN- or rap-induced autophagy was combined with the lack of ULK1, the intensity of autophagy became drastically decreased compared to single rap or SFN treatments (see the lower LC3 II and higher p62 levels in Figure 4B).

These results suggest that SFN induces autophagy through the inhibition of mTORC1, like rapamycin, rather than in a PKA-dependent manner, and it also assumes that ULK1 is required for SFN-induced autophagy.

### 2.4. Excessive Level of Permanent ER Stress Can Be Postponed with SFN-Induced Autophagy

Recently, we have identified various natural compounds (such as resveratrol and EGCG) that were able to disrupt the balance of the mTORC1-AMPK pathway and thus induced autophagy-dependent survival during ER stress [26,27]. Since SFN was able to induce autophagy, too, we investigated whether SFN had a positive effect on cell survival during excessive levels of permanent ER stress induced by TG.

**Figure 5:**
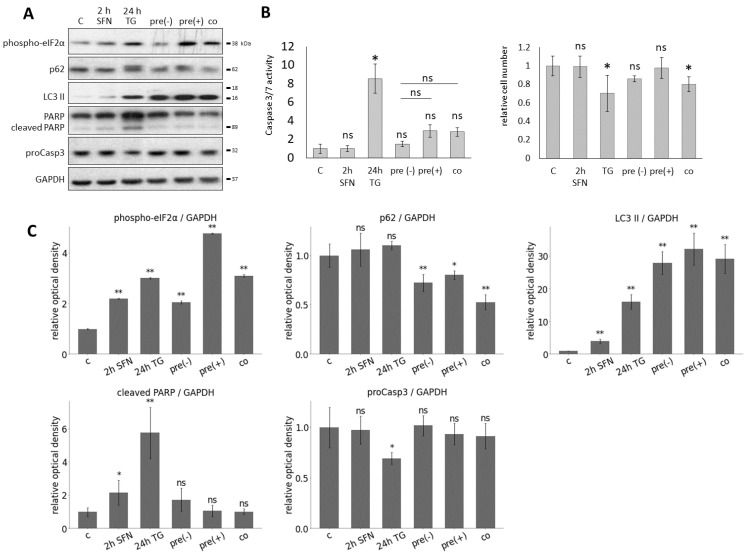
The effect of prolonged ER stress combined with sulforaphane treatment. HEK293T cells were treated with 15 μ M SFN for 2 h, 100 nM TG for 24 h in the following combinations: pre-treatment without washout of SFN (pre(+)) and with washout of SFN (pre(−)) and co-treatment (co). By the pre-treatment, the SFN was added 2 h before the TG treatment, and by the co-treatment, SFN and TG were added at the same time point. (**A**) The markers of ER stress (phospho-eIF2 α ), autophagy (p62, LC3 II), and apoptosis (cleaved PARP, proCasp3) were followed by immunoblotting. GAPDH was used as a loading control. (**B**) The caspase 3/7 activity (left panel) and the number of viable cells (right panel) were denoted. (**C**) Densitometry data represent the intensity of phospho-eIF2 α , p62, LC3 II, cleaved PARP, and proCasp3 normalized for GAPDH. Error bars represent standard deviation. Asterisks indicate statistically significant differences from the control: *ns*—non significant; *—*p* < 0.05; **—*p* < 0.01.

It is well-known that TG disrupts the calcium storage of ER in the cells [64]. To explore the effect of SFN on ER stress, HEK293T cells were either pre-treated with SFN (15 μ M, 2 h) followed by the addition of TG (100 nM, 24 h), or SFN was added simultaneously with TG (co). Two types of pre-treatments were performed: one where SFN was washed out after 2 h (pre(−)), and the other one where SFN was not washed out after the 2 h pre-treatment (pre(+)) (Figure 5). To investigate whether SFN was able to maintain cell viability in response to ER stress, the relative number of viable cells was measured during treatments (Figure 5B). The presence of SFN in pre(−)- and pre(+)-treatments kept cell viability at a level similar to the control and appeared to work better than the co-treatment (Figure 5B).

To detect the effect of SFN with respect to ER stress, autophagy and apoptosis markers were followed during the combined treatments by immunoblotting (Figure 5A). Based on the phosphorylation of eIF2 α , ER stress was observed upon treatment in each case. When 24 h long TG treatment was combined with SFN as a pre-treatment (pre(−) and pre(+)) or a co-treatment, a remarkably high level of LC3II and the decrease in p62 indicated that autophagy remained still active. This is confirmed by the fact that neither a decrease in proCasp3 nor a cleavage of PARP was observed, so the apoptosis was inactive. (Figure 5A). We also performed a caspase 3/7 activity (ApoOne) assay to confirm our immunoblot results. Based on the caspase 3/7 activity, all three combined treatments (pre(−), pre(+), co) significantly reduced apoptosis activation compared to 24 h TG treatment (Figure 5B).

**Figure 6:**
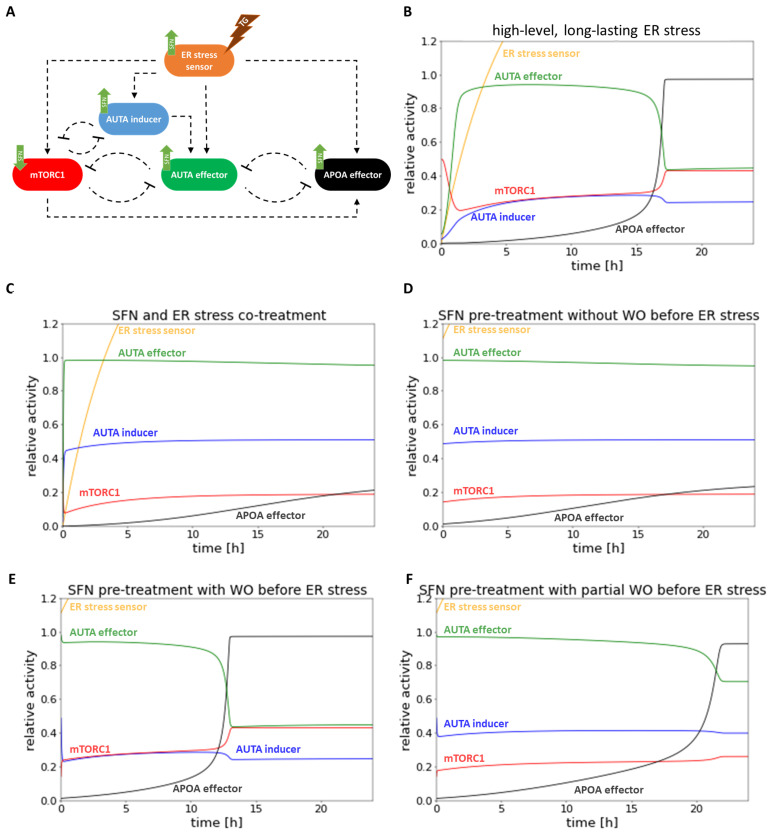
The computer simulations of the prolonged combined treatment in time. (**A**) The wiring diagram of endoplasmic reticulum (ER) stress-response mechanism. (**B**) The high-level, long-lasting ER stress (parameter setting for the simulation: stress = 100). (**C**) SFN (parameter setting for the simulation: SFN = 1, stress = 10) and high-level, long-lasting ER stress (parameter setting for the simulation: stress = 100) co-treatment. (**D**) SFN pre-treatment (parameter setting for the simulation: SFN = 1, stress = 10) without washout (WO) before high-level, long-lasting ER stress (parameter setting for the simulation: stress = 100). (**E**) SFN pre-treatment (parameter setting for the simulation: SFN = 1, stress = 10) with washout (WO) before high-level, long-lasting ER stress (parameter setting for the simulation: stress = 100). (**F**) SFN pre-treatment (parameter setting for the simulation: SFN = 1, stress = 10) with partial washout (WO) parameter setting for the simulation after the wash SFN = 0.5, stress = 5 by the SFN treatment) before high-level, long-lasting ER stress (parameter setting for the simulation: stress = 100). The relative activity of the ER stress sensor, AUTA inducer, mTORC1, APOA effector, and AUTA effector are plotted in time.

With computer simulations, the dynamic behavior of the regulatory network was also studied (Figure 6). Under high-level, long-lasting ER stress, we first saw the activation of autophagy in parallel with the inactivation of mTORC1. However, as the simulation time increased, autophagy was inactivated while mTORC1 activity increased, and apoptosis was switched on (Figure 6B).

Then, ER stress and SFN treatment were combined and followed by computer simulations. According to our experimental data, no activation of apoptosis was observed, while autophagy became permanently active in both SFN-TG co-treatment (Figure 6C) and SFN pre-treatment without washing it out (Figure 6D), too. Interestingly, we observed a difference between experimental and theoretical results in the case of complete washout of SFN pre-treatment (Figure 6E,F) followed by the addition of TG. Specifically, the time series result of complete washout of SFN was not able to keep active autophagy anymore; rather, it resulted in rapid activation of apoptosis upon simulating ER stress. However, when we assumed that the washing out of SFN was not complete (only 50%), autophagy remained active longer, and apoptosis could be activated much later. This time series result was much closer to the experimental result we presented above (Figure 5).

Based on our systems biology analysis, SFN can induce autophagy-dependent survival, whereas the activation of apoptosis might be delayed in the presence of prolonged ER stress.

### 2.5. The Negative Effect of Acute ER Stress Can Be Delayed with Pre-Treatment of SFN

**Figure 7:**
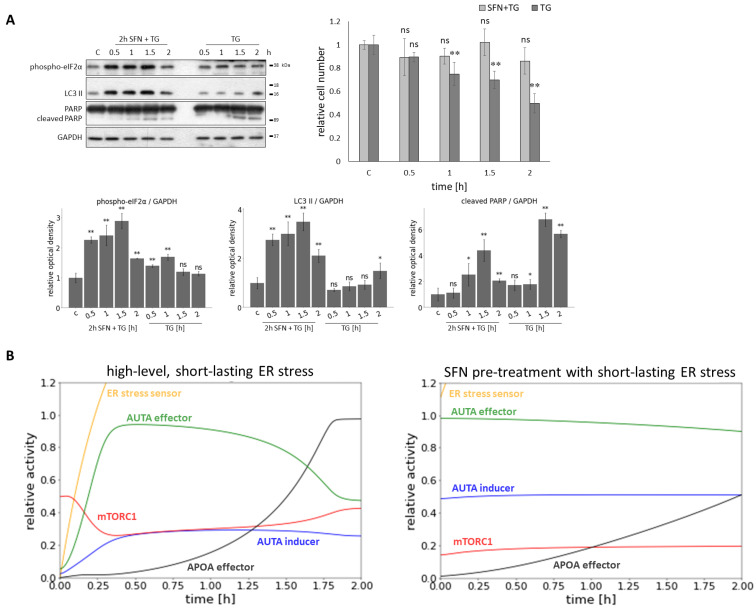
The effect of acute ER stress combined with sulforaphane treatment. HEK293T cells were treated with 25 μ M SFN for 2 h and 10 μ M TG for 2 h. In the pre-treatment, the SFN was added 2 h before the TG treatment. (**A**) The markers of ER stress (phospho-eIF2 α ), autophagy (LC3 II), and apoptosis (cleaved PARP) were followed by immunoblotting. GAPDH was used as a loading control (left panel). The number of viable cells was denoted (right panel). Densitometry data represent the intensity of (phospho-eIF2 α ), LC3 II, and cleaved PARP normalized for GAPDH (lower panel). Error bars represent standard deviation. Asterisks indicate statistically significant differences from the control: *ns*—non significant; *—*p* < 0.05; **—*p* < 0.01. (**B**) The computer simulations of the acute ER stress (parameter setting for the simulation: stress = 1500) (left panel) and SFN pre-treatment (parameter setting for the simulation: SFN = 1, stress = 10) before the acute ER stress parameter setting for the simulation: stress = 1500) (right panel) in time. The relative activity of the ER stress sensor, AUTA inducer, mTORC1, APOA effector, and AUTA effector are plotted in time.

Although our results mostly related to long-term ER stress, the question was raised as to what the effect of SFN can be in the case of acute severe ER stress (Figure 7).

To study the effect of SFN during acute ER stress, SFN was used as a 2 h pre-treatment at a concentration of 25 μ M in HEK293T cells. TG was used as an ER stressor at a concentration of 10 μ M, with treatments lasting 0.5, 1, 1.5, and 2 h (Figure 7A). SFN pre-treatment improved cell survival (Figure 7A) by maintaining the number of viable cells at around 80% or above even after 2 h of ER stress. In contrast, without pre-treatment, the number of viable cells showed a greater decrease. Next, the effect of pre-treatment was also assessed by immunoblotting of the key proteins ((phospho-eIF2 α ), LC3 II, cleaved PARP) (Figure 7A). Based on eIF2 α phosphorylation, ER stress developed with increasing treatment duration. LC3 II was significantly increased during SFN+TG combined treatment, indicating intense autophagy. Without pre-treatment, a smaller increase in LC3 II was observed only after 2 h of treatment, so autophagy occurred later in that case. As stress progressed, a slight increase in PARP cleavage was detected in the combined treatment, but this decreased by 2 h. Without pre-treatment, the PARP cleavage increased, indicating activation of apoptotic processes that were more intense than in the pre-treatment condition (Figure 7A).

Our model showed that under acute ER stress, mTORC1 initially became inactive while autophagy was activated. However, mTORC1 activity increased, and therefore, apoptosis could be switched on after the inactivation of autophagy (Figure 7B). When SFN pre-treatment was applied before acute ER stress, autophagy remained active until the end of treatment, whereas activation of mTORC1 and apoptosis occurred later (Figure 7B).

Taken together, when sulforaphane is used as a pre-treatment before acute ER stress, autophagy becomes more active than without sulforaphane, and apoptosis turns on at a later stage.

## 3. Discussion

Cellular life-and-death decision is one of the most important tasks of a living organism to answer the drastic effects of various external and internal stimuli (such as endoplasmic reticulum stress, starvation, etc.). Recently, we have developed a simple mathematical model that was able to describe the important regulatory connections of the control network, claiming the importance of the double-negative feedback loops between the surviving and cell-death mechanisms [23,25]. We supposed that these feedback loops guaranteed the switch-like characteristic of the response corresponding to the level of cellular stress [23,25]. In the last couple of years, we also confirmed the important roles of various natural compounds (i.e., resveratrol, EGCG) in extending autophagy-dependent cell survival by postponing ER stress-induced apoptotic cell death. Our dynamical analysis using systems biological methods suggests that these components used in the right concentrations and for the right length of time may even be of medical relevance in the future [23,26,27].

Recently, another natural substance, called sulforaphane (SFN), found in cruciferous vegetables (i.e., broccoli and cabbage), has been increasingly used in medical circles. SFN is an organosulfur compound, and it is produced by enzymes when the plant becomes damaged [16,65]. Although there is a great deal of scientific evidence that SFN is medically beneficial for the patient (i.e., it reduces the symptoms of autism, contributes to preventing cancer development, and has an anti-oxidant effect) [66], direct molecular biology results are sometimes contradictory. Specifically, SFN treatment can increase the expression of ER stress genes in lens cells. Meanwhile, autophagy-dependent cell death is detected [38]. Results are also divided on whether SFN really has a positive or negative effect on autophagy and apoptosis. On the one hand, SFN could inhibit autophagy by stopping the fusion between autophagosome and autolysosome in esophageal squamous cell carcinoma cells [39] while on the other hand, SFN promoted the autophagy processes in some cases (e.g., in pancreatic carcinoma cells and prostate cancer cells) [67,68]. Moreover, SFN was able to activate apoptosis, e.g., in gastric cancer cells through p53 [59] and in nasopharyngeal cancer cells [69]. This leads directly to the question of what the biological significance of this might be. Therefore, to investigate the dual role of SFN further, we carried out a systems biological analysis where both experimental and theoretical methods were used.

We have taken our previously developed model as the starting point for our analysis here. In this simple model, ER stress was induced by a so-called ER stress sensor, which was able to turn on mTORC1, autophagy, and apoptosis, respectively (Figure 1). According to our previous experimental data [18,23], the double-negative feedback loops between mTORC1 and autophagy and also between autophagy and apoptosis in this model guaranteed that both short- and long-lasting low level of ER stress turned on autophagy, meanwhile apoptotic cell death remained inactive (Figure S3). However, excessive levels of ER stress resulted in transient activation of autophagy, but later, it was downregulated, and the cell committed suicide via apoptosis (Figure 7A,B). Taking into account previously published experimental results about SFN, first, we assumed that SFN induced ER stress sensor, autophagy, and apoptosis, while it had a negative effect on mTORC1 (Figure 1).

The effect of SFN on HEK293T cells was then investigated as a function of both concentration and time. The diagram of the relative number of viable cells suggested that short SFN treatment did not induce cell death even at high concentrations (Figure 2A and Figure S1). However, longer treatments have already shown a drastic decrease in cell number at higher SFN concentrations (Figure 2A and Figure S1). This confirmed that cells were sensitive to the amount of SFN and the length of treatment. From this, we conclude that in the case of medical use, the treatment conditions should be set very thoroughly and fully, and maybe individualized.

The next question that we have tried to answer here is what specifically could cause cell death during SFN treatment. Our computer simulations suggested that a low level of SFN induced autophagy, while the high level of SFN turned on both autophagy and apoptosis (Figure 2B,C and Figure S3). These theoretical analyses were supported by immunoblot data (Figure 3 and Figure S2). We confirmed that SFN induced ER stress even at short treatment, but this effect was not fatal for the cell. Moreover, at low concentrations or short SFN treatments, autophagy induction coupled with ER stress did not reduce cell viability. In contrast, at longer treatments, when cell death was detected, apoptosis was already induced. Interestingly, autophagy appeared to remain active in addition to apoptosis (Figure 2C and Figure 3B,C). Although there is an antagonism between autophagy and apoptosis based on a double-negative feedback loop, the positive effect of SFN was so strong on them that both became activated. Therefore, our result supports the previously published contradictory data on how SFN affects autophagy and apoptosis. These data further confirm that cellular systems are really sensitive to the amount and duration of SFN treatment.

Some experimental data suggested that SFN promoted autophagy via direct inactivation on mTORC1 and also via enhancing AMPK [39,40,70], which is an inhibitor of mTORC1, therefore generating an indirect negative effect on mTORC1, while some data supposed that mTOR signaling pathway was not involved in SFN-induced autophagy [37]. Here, we presented that SFN-induced autophagy required ULK1 (the key component of the autophagy activator complex) and indeed acted via the mTORC1 pathway (Figure 4).

Since ER stress is present in many diseases and it has been previously demonstrated that various natural agents can delay ER stress-induced cell death, it is immediately questionable what effect SFN might have in TG-induced ER stress. For this treatment, we applied SFN at a concentration that did not yet reduce cell viability but induced autophagy. To investigate its effect on HE293T cells, SFN was added simultaneously with TG (co). Meanwhile, two different pre-treatments were also carried out: one where SFN was washed out before the addition of TG (pre(−)), and the other one where SFN was not washed out after pre-treatment (pre(+)) (Figure 5). Our theoretical results suggested that (co) and (pre(+)) treatments could increase cell viability by delaying the activation of apoptosis. Meanwhile, autophagy remained active. However, in the case of (pre(−)) treatment, autophagy turned off quickly, and apoptosis was not postponed at all (Figure 6A–E). Although up to this point, our model correctly described the experimental results, thus supporting them, we have already experienced a difference between them here. Specifically, all three types of experiments suggested that cell viability did not decrease significantly, autophagy was hyper-activated, while apoptotic cell death was postponed. The discrepancy between our experimental and theoretical results could be resolved if we assumed that in the case of (pre(−)) treatment, the SFN was not washed out totally before the ER stressor was induced in the cells (Figure 6F). Our theoretical analysis claims that some SFN is essential for autophagy-dependent survival to win against apoptotic cell death. In the near future, we would like to experimentally investigate the speed and kinetics of SFN excretion from the cells after they have been washed out. Interestingly, Rajendran et al. found that SFN was cleared of the body within 24 h; however, SFN protection from cancer was induced even when the substance was eliminated from the body [71]. Since cells are particularly sensitive to the length and concentration of SFN treatment in terms of output (namely, whether they survive or die), it is definitely necessary to investigate more thoroughly how SFN is stored and cleared from cells. Knowing this, it will be possible to use the SFN treatment for medical purposes much more precisely and efficiently.

Although persistent ER stress is observed in most diseases, in some cases, the acute ER stress response of cells may also be important [72,73]. Therefore, we also carried out transient ER stress pre-treated with/without low level of SFN (Figure 7). Although short ER stress with high concentration induced autophagy-dependent survival followed by apoptotic cell death, SFN pre-treatment was able to maintain autophagy and postpone apoptosis. These results further confirm that SFN might have a therapeutic role by increasing cell viability via autophagy, meanwhile, delaying apoptotic cell death.

In the various clinical trials, SFN has been used orally in a range of 9.9–847 μ mol/ person/day [65], which is relatively wide. Thus, based on recent research, the main challenges to the use of sulforaphane are its highly variable bioavailability and rapid metabolism and elimination from the body [65,74]. For this reason, the precise determination of treatment concentrations is critical, and the best effect is probably achieved when therapies are personalized and tailored to the specific disease (or even the tissue involved), in which our model can be of great help. By adequately fitting the model, rapid selection of treatment concentrations and times will be possible in the light of preliminary outcomes and be successfully used in medical treatments.

## 4. Materials and Methods

### 4.1. Materials

Thapsigargin (TG) (Sigma-Aldrich, St. Louis, MO, USA; T9033), rapamycin (Sigma-Aldrich, R0395), sulforaphane (Sigma-Aldrich, S4441) and H89 (Adipogen, Fuellinsdorf, Switzerland; AG-CR1-0002) were purchased. All other chemicals were of reagent grade.

### 4.2. Cell Culture and Maintenance

As a model system, a human embryonic kidney (HEK293T, ATCC, CRL-3216; Manassas, VA, USA) cell line was used. It was maintained in DMEM (Life Technologies, Carlsbad, CA, USA; 41965039) medium supplemented with 10% fetal bovine serum (Life Technologies, 10500064) and 1% antibiotics/antimycotics (Life Technologies, 15240062). Culture dishes and cell treatment plates were kept in a humidified incubator at 37 °C in 95% air and 5% CO ${\;}_{2}$ .

### 4.3. SDS-PAGE and Western Blot Analysis

Cells were harvested and lysed with 20 mM Tris, 135 mM NaCl, 10% glycerol, 1% NP40, pH 6.8. The protein content of cell lysates was measured using Pierce BCA Protein Assay (Thermo Fisher Scientific, Waltham, MA, USA; 23225). During each procedure, equal amounts of protein were used. SDS-PAGE was carried out using Hoefer miniVE (Amersham). Proteins were transferred onto Millipore 0.45 μ m PVDF membrane. Immunoblotting was performed using TBS-Tween (0.1%), containing 5% non-fat dry milk, 1% bovine serum albumin (Sigma-Aldrich, A9647) or gelatine buffer (Sigma-Aldrich, G8327) for blocking membrane and for antibody solutions. Loading was controlled by developing membranes for GAPDH or dyed with Ponceau S in each experiment. For each experiment, at least three independent measurements were carried out. The following antibodies were applied: antip62 (Cell Signaling, Danvers, MA, USA; 5114S), antiLC3B (SantaCruz, Santa Cruz, CA, USA; sc-16755), antiCaspase3 (SantaCruz, sc-7272), antiPARP (Cell Signaling, 9542S), antiULK1 (Cell Signaling, 8054S), anti-phospho-p70S6K (Cell Signaling, 9234S), antip70S6 (SantaCruz, sc-9202), anti-phospho-eIF2 α (Cell Signaling, 9721S), and antiGAPDH (Santa Cruz, 6C5), HRP conjugated secondary antibodies (Cell Signaling, 7074S, 7076S). The bands were visualized using a chemiluminescence detection kit (Thermo Fisher Scientific, 32106).

### 4.4. RNA Interference

RNA interference experiments were performed using Lipofectamine RNAi Max (Invitrogen Thermo Fisher Scientific Inc., Waltham, MA, USA) in GIBCO™Opti-MEM I (GlutaMAX™-I) Reduced-Serum Medium liquid (Invitrogen) and 20 pmol/mL siRNA. The siULK1 oligonucleotides were purchased from Ambion (Ambion Thermo Fisher Scientific Inc., Waltham, MA, USA; AM16708). 200,000 HEK293T cells were incubated at 37 °C in a CO ${\;}_{2}$ incubator in an antibiotic-free medium for overnight, then the RNAi duplex-Lipofectamine™RNAiMAX (Invitrogen, 13778-075) complexes were added to the cells for overnight. Then, a fresh medium was added to the cells, and the appropriate treatment was carried out. To check the efficiency of ULK1 silencing, a Western blot was used with a ULK1 monoclonal antibody (Cell Signaling, 8054S).

### 4.5. Apo-ONE® Homogeneous Caspase-3/7 Assays

The Apo-ONE® Homogeneous Caspase-3/7 Assay (Promega, Madison, WI, USA; G7790) was used for photometric assays of caspase3 and caspase7 activity. The reagent consists of a mixture of a substrate (rhodamine 110 bis-(N-CBZ-l-aspartyl-l-glutamyl-l-valyl-aspartic acid amide) (Z-DEVD-R110)) and a cell lysis/activity buffer. HEK293T cells were seeded on a black optical well plate to avoid light scattering into adjacent wells. After treatment, an equal amount of Apo-ONE® reagent was added to each well. Then, the well plate was shaken for 30 s at 300 rpm. After 3 h of incubation at room temperature, photometric measurements were performed with the CLARIOstar® Plus Microplate Reader at 485/520 nm, using the enhanced dynamic range tool. The fluorescence signal obtained is directly proportional to the activity of the caspase enzymes, allowing for an inference of the extent of apoptosis.

### 4.6. Cell Viability Assay

The relative number of viable cells was calculated by Luna Automata Cell Counter. The cells were stained with trypan blue dye. Cell viability was detected using CellTiter-Blue® Cell Viability Assay (Promega, G8080). Cells were grown and treated on 96-well plates and were incubated with resazurin for 2 h at 37 °C. Absorbance was measured at 620 nm and expressed in arbitrary units, being proportional to cell toxicity. For each of these experiments, at least three parallel measurements were carried out.

### 4.7. Mathematical Modelling

The dynamic behavior of the control network was described by nonlinear ordinary differential equations (ODEs) [75,76,77], resulting in a multi-parameter first-order differential equation system. The analysis of the dynamic simulations was performed using the XPP-AUT 8.0 program, which is available free of charge at the website: https://sites.pitt.edu/phase/bard/bardware/xpp/xpp.html (accessed on 30 November 2023) [76,77]. In the simulations (in signal response curve and in time-course simulation, too), only the parameters of the treatments were changed in the model fitted based on the experimental results. For details, see the Supplementary Information [78,79,80,81,82,83].

### 4.8. Statistics

For densitometry analysis, Western blot data were acquired using ImageJ software. The relative band densities were shown and normalized to an appropriate total protein or GAPDH band used as a reference protein. The control (c) contains untreated cells incubated for the same time as the treated cells, and the data were always normalized to the untreated control. For each of the experiments, three independent measurements were carried out. Results are presented as mean values ± S.D. and were compared using ANOVA with Tukey’s multiple comparison post hoc test. Asterisks indicate statistically significant differences from the appropriate control: *ns*—non significant; *—*p* < 0.05; **—*p* < 0.01.

## Data Availability

Data is contained within the article and Supplementary Material.

## Supplementary Materials

The following supporting information can be downloaded at: www.mdpi.com/xxx/s1.

## Abbreviations

The following abbreviations are used in this manuscript:

mTORC1	mammalian target of rapamycin complex 1
SFN	sulforaphane
rap	rapamycin
TG	thapsigargin
ER	endoplasmic reticulum
AUTA	autophagy
APOA	apoptosis
