# Supplementary material for: The Dual Role of Sulforaphane-Induced Cellular Stress—A Systems Biological Study
Source: Int J Mol Sci. 2024 Jan 19;25(2):0. doi: 10.3390/ijms25021220 (PMC11154497; doi:10.3390/ijms25021220)
Supplement: Supplementary file 1 [file ijms-25-01220-s001.zip › ijms-2763473-supplementary.pdf]

Supplementary Information
"The dual role of sulforaphane-induced cellular stress – a systems biological study"

1 Describing the theoretical analysis

In this section, we briefly describe the mathematical approach used to study the effect of sulforaphane. By combining the components and interactions reported in the literature, a system-level model can be constructed. Such a network can be translated into a set of mathematical equations that describe how the concentration/activity of each component in the network changes over time. The rate of change of a component is described by an ordinary differential equation (ODE) based on biochemical reaction kinetics (see equation below). Each biochemical reaction is represented on the right-hand side of the ODE as an expression for the component involved in the reaction (Strogatz (1994); Tyson et al. (2001)). Each reaction in the network can be described by the law of mass action or Michaelis-Menten kinetics (Goldbeter and Koshland (1981); Segel (1975); Tyson et al. (2003)).
The general differential equation describing the time variation of protein X_a consists of two parts: production and consumption terms.

$$\frac{dX_a}{dt} = k_s + k_a * (X_t - X_a) - (k_d + k_i) * X_a \tag{1}$$

Where:
X_a – concentration of active X
X_t – total concentration of X
k_s – synthesis rate constant of X
k_a – activation rate constant of Xa
k_d – degradation rate constant of X
k_i – inactivation rate constant of Xa

Production is expressed as protein synthesis and/or activation, while consumption is expressed as protein degradation and/or inactivation. In general, synthesis, degradation, binding and dissociation reactions can be described by mass action kinetics, while protein activity can be described by mass action or Michaelis-Menten kinetics (Tyson et al. (2003, 2002)). For example, if the protein activity is controlled by covalent modification involving multi-site phosphorylations, Michaelis-Menten kinetics provides a good approximation of the process (Ferrell (1996); Kapuy et al. (2009)). To solve ODEs, the parameters (rate constants, Michaelis constants) and initial conditions must be specified. Due to the non-linear nature of biological processes, ODEs are difficult to solve analytically and therefore the equations must be solved numerically. The equations can be solved using different numerical integration methods, which have been implemented as solvers in a number of freely available computer software.
The solution of a set of non-linear ODEs gives the time evolution of the protein concentration/activity, the so-called **time courses**. Furthermore, ODEs can be solved to determine the input-output relationship called **signal response curves** (Kaplan and Glass (1995); Strogatz (1994); Tyson et al. (2003)). The input is the

signal strength, which is varied to achieve a steady-state behaviour of the control system. This helps to capture the qualitative changes in the behaviour of the system. For example, the system behaviour can become abrupt and discontinuous when the signal strength is increased from a low value to a high value. The point where such a qualitative change occurs in the system is defined as the bifurcation point (Strogatz (1994)).

In this work, temporal profiles and signal response curves were numerically calculated using *XPP-AUT*. All the simulations presented in the text are based on the following XPP codes. The rate constants (k) have the dimension of min^{-1} and Michaelis constants (J) are dimensionless. The protein levels/activities are given in arbitrary units (a.u). The starting parameter set was able to refer to physiological conditions. The parameter values were perturbed to capture all possible qualitative behaviours that the given network can exhibit.

1.1 The code for time course simulations

```
# differential equations
# ERS represents the active form of endoplasmic reticulum stress sensor
ERS' = kaers*STRESS*(ERST-ERS) - kiers*ERS

# Ind represents the active form of autophagy inducer
Ind' = (kaamp + alfa*SFN + kaamp'*ERS)*(IndT-Ind) - (kiamp + kiamp'*mTOR)*Ind

# mTOR represents the active form of mTORC1
mTOR' = (kamtor + kamtor'*ERS)*(mTORT-mTOR) - (kimtor + beta*SFN +
kimtor'*Auta + kimtor"*Ind)* mTOR

# Apoa represents the active form of apoptosis effector
Apoa' = (kaap + gamma*SFN + kaap'*ERS + kaap"*mTOR)*(Apot-Apoa)/(Jap
+ Apot-Apoa) - (kiap + kiap'*Auta)*Apoa/(Jap + Apoa)

# Auta represents the active form of autophagy effector
Auta' = (kaau + delta*SFN + kaau'*ERS + kaau"*Ind)*(Autt-Auta)/(Jau + Autt-
Auta) - (kiau + kiau'*Apoa + kiau"*mTOR)*Auta/(Jau + Auta)

# parameters
# 2 hours high ER stress: stress= 1500
# 24 hours high ER stress: stress= 100
# 2 hours low ER stress: stress= 50
# 24 hours low ER stress= 25
# low SFN treatment: SFN= 1, stress= 10
# high SFN treatment: SFN= 80, stress= 10
p stress=0
p kaers=0.002, kiers=0.01, ERST=2
p kaamp=1, kaamp'=5, kiamp=10, kiamp'=50, IndT=1
p kamtor=5, kamtor'=5, kimtor=3, kimtor'=30, kimtor"=10, mTORT=1
p Jap=0.01, kaap=1, kaap'=5, kaap"=1, kiap=5, kiap'=6.5, Apot=1
p Jau=0.4, kaau=0.5, kaau'=10, kaau"=5, kiau=1, kiau'=20, kiau"=5, Autt=1
```

p SFN=0, alfa=10, beta=25, delta=20, gamma=0.125

done

1.2 The code for simulating signal response curves

```
# differential equations
# Apoa represents the active form of apoptosis effector
Apoa' = (kaap + gamma*SFN + kaap'*ERS + kaap"*mTOR)*(Apot-Apoa)/(Jap + Apot-Apoa) - (kiap + kiap'*Auta)*Apoa/(Jap + Apoa)

# Auta represents the active form of autophagy effector
Auta' = (kaau + delta*SFN + kaau'*ERS + kaau"*Ind)*(Autt-Auta)/(Jau + Autt-Auta) - (kiau + kiau'*Apoa + kiau"*mTOR)*Auta/(Jau + Auta)

# steady state function
# ERS represents the active form of endoplasmic reticulum stress sensor
ERS = kaers*STRESS*ERST/( kaers*STRESS + kiers)

# Ind represents the active form of autophagy inducer
Ind = (kaind + alfa*SFN + kaind'*ERS)* IndT/(kaind + alfa*SFN + kaind'*ERS + kiind + kiind'*mTOR)

# mTOR represents the active form of mTORC1
mTOR = (kamtor + beta*SFN + kamtor'*ERS)*mTORT/(kamtor + beta*SFN + kamtor'*ERS + kimtor + kimtor'*Auta + kimtor"*Ind)

# parameters
# 2 hours high ER stress: stress= 1500
# 24 hours high ER stress: stress= 100
# 2 hours low ER stress: stress= 50
# 24 hours low ER stress= 25
# low SFN treatment: SFN= 1, stress= 10
# high SFN treatment: SFN= 80, stress= 10
p stress=0
p kaers=0.002, kiers=0.01, ERST=2
p kaind=1, kaind'=5, kiind=10, kiind'=50, IndT=1
p kamtor=5, kamtor'=5, kimtor=3, kimtor'=30, kimtor"=10, mTORT=1
p Jap=0.01, kaap=1, kaap'=5, kaap"=1, kiap=5, kiap'=6.5, Apot=1
p Jau=0.4, kaau=0.5, kaau'=10, kaau"=5, kiau=1, kiau'=20, kiau"=5, Autt=1
p SFN=0, alfa=10, beta=25, delta=20, gamma=0.125

done
```

2 Supplementary figures

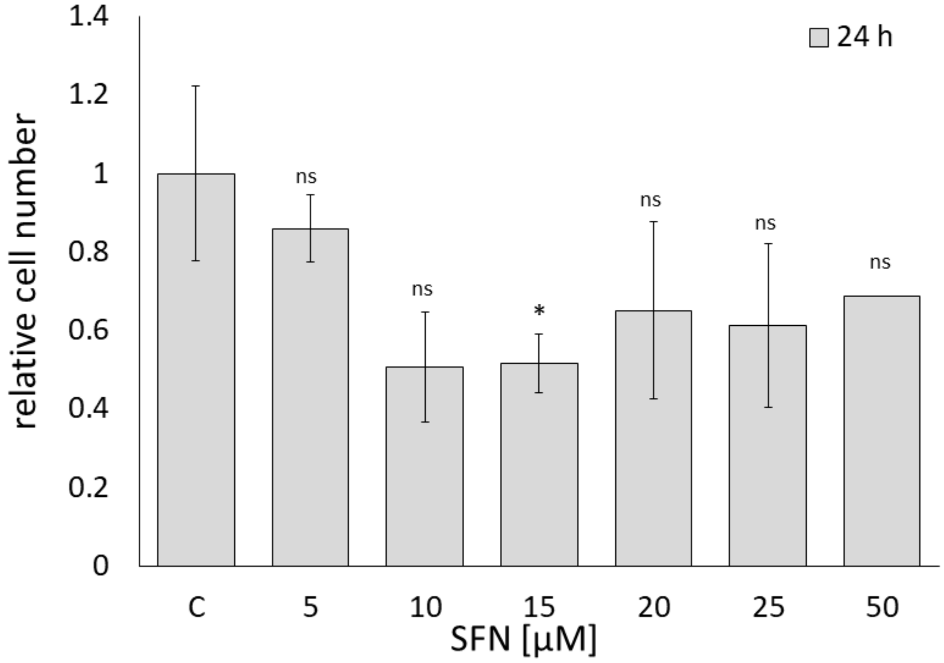

Figure S1: The time- and concentration-dependent effect of the sulforaphane on the viability of the cells. HEK293T cells were treated with 5, 10, 15, 20, 25 and 50 µM SFN for 24 hours meanwhile the relative number of viable cells was denoted. Error bars represent standard deviation, asterisks indicate statistically significant differences from the control: * - $p < 0.05$; ** - $p < 0.01$.

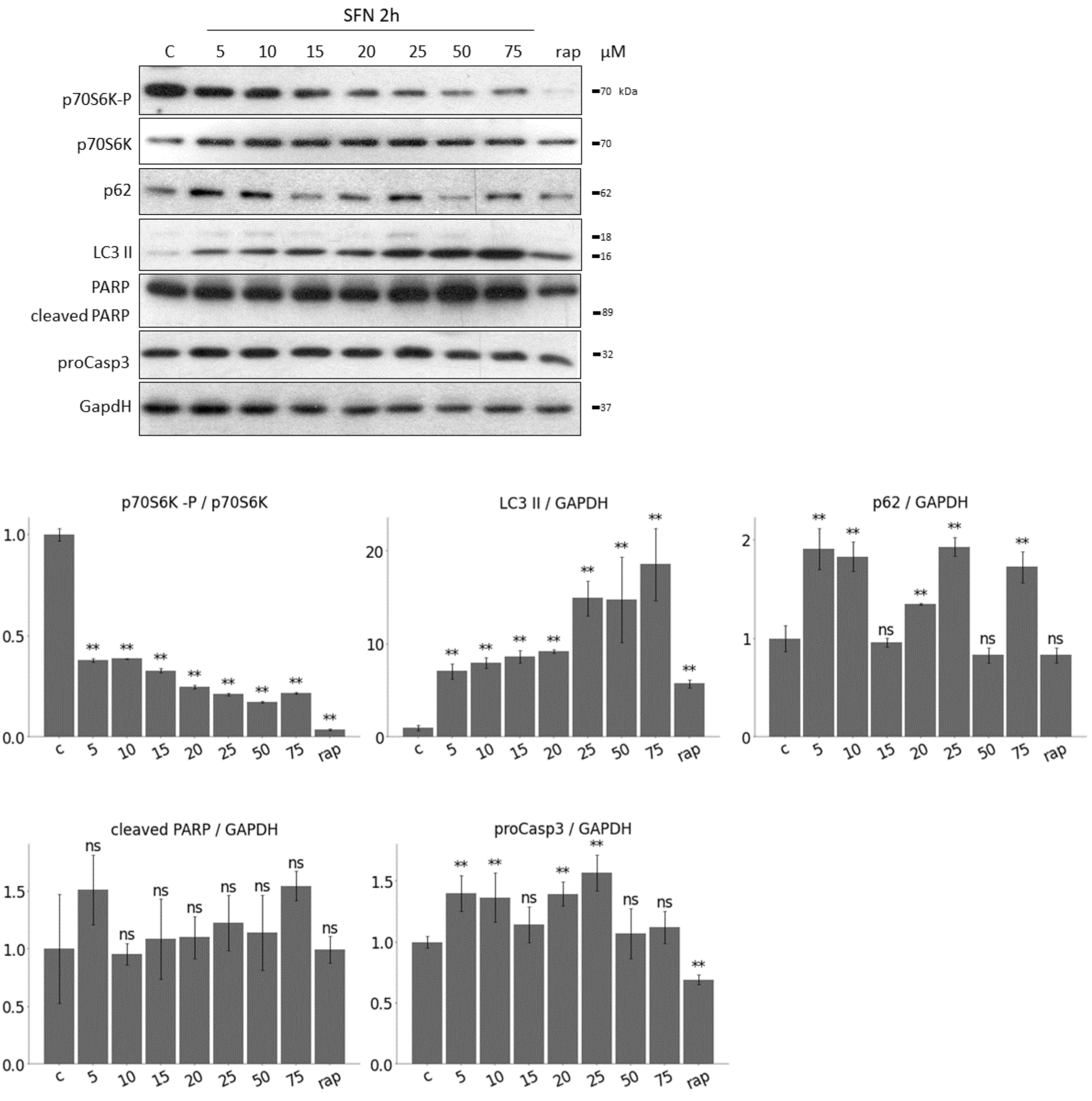

Figure S2: The time- and concentration-dependent effect of the sulforaphane on the members of the control network – with a positive control. HEK293T cells were treated with 5, 10, 15, 20, 25, 50, and 75 µM SFN for 2 hours; and 100 nM rapamycin (rap) for 2 hours. The markers of mTORC1 (p70S6K-P), autophagy (p62, LC3 II), and apoptosis (cleaved PARP, proCasp3) were followed by immunoblotting. GAPDH was used as a loading control (left panel). Densitometry data represent the intensity of p70S6K-P normalized for total level of p70S6K, p62, LC3 II, cleaved PARP and proCasp3 normalized for GAPDH. Error bars represent standard deviation, asterisks indicate statistically significant differences from the control: * - $p < 0.05$; ** - $p < 0.01$.

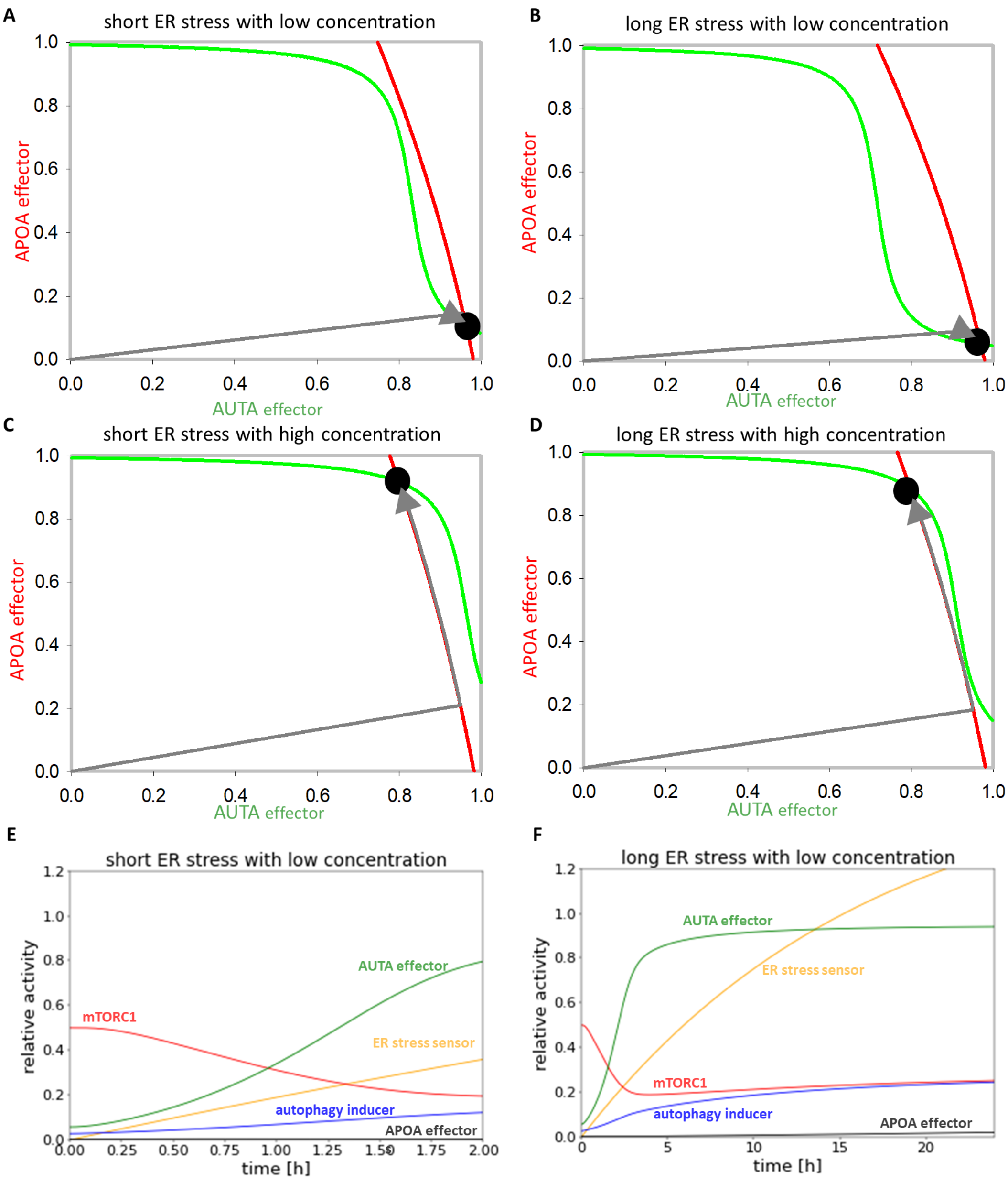

Figure S3: The computer simulations of additional treatments. **a**) Phase plane diagram of the short and low ER stress (stress=50). **b**) Phase plane diagram of the prolonged low ER stress (stress=25). **c**) Phase plane diagram of the short and severe ER stress (stress=1500). **d**) Phase plane diagram of the prolonged severe ER stress (stress=100). The balance curves of autophagy effector (green curve) and apoptosis effector (red curve) are plotted. Stable steady states are visualized with black dots. **e**) The computational simulations of the short and low ER stress (stress=50) in time. **f**) The computational simulations the prolonged low ER stress (stress=25) in time. The relative activity of ER stress sensor, autophagy inducer, mTORC1, apoptosis effector, and autophagy effector are plotted in time.

References

Ferrell, J. E., J. (1996). Tripping the switch fantastic: how a protein kinase cascade can convert graded inputs into switch-like outputs. *Trends Biochem Sci 21*(12), 460–6.

Goldbeter, A. and J. Koshland, D. E. (1981). An amplified sensitivity arising from covalent modification in biological systems. *Proc Natl Acad Sci U S A. 78*(11), 6840–4.

Kaplan, D. and L. Glass (1995). *Understanding Nonlinear Dynamics.* New York: Springer-Verlag.

Kapuy, O., D. Barik, M. R. Sananes, J. J. Tyson, and B. Novak (2009). Bistability by multiple phosphorylation of regulatory proteins. *Prog Biophys Mol Biol. 100*(1-3), 47–56. Epub 2009 Jun 11.

Segel, I. H. (1975). *Enzyme kinetics behavior and analysis of rapid equilibrium and steady state enzyme systems.* Wiley.

Strogatz, S. H. (1994). *Nonlinear Dynamics and Chaos.* Reading, MA: Addison-Wesley Co.

Tyson, J. J., K. Chen, and B. Novak (2001). Network dynamics and cell physiology. *Nature Rev. Mol. Cell Biol. 2*, 908–916.

Tyson, J. J., K. C. Chen, and B. Novak (2003). Sniffers, buzzers, toggles and blinkers: dynamics of regulatory and signaling pathways in the cell. *Current Opinion in Cell Biology 15*(2), 221–231.

Tyson, J. J., A. Csikasz-Nagy, and B. Novak (2002). The dynamics of cell cycle regulation. *BioEssays 24*, 1095–1109.
